# Anterior Incisura Fibularis Corner Landmarks Can Safely Validate the Optimal Distal Tibiofibular Reduction in Malleolar Fractures—Prospective CT Study

**DOI:** 10.3390/diagnostics13152615

**Published:** 2023-08-07

**Authors:** Meletis Rozis, Dimitrios Zachariou, Michalis Vavourakis, Elias Vasiliadis, John Vlamis

**Affiliations:** 3rd Orthopedic Department, University of Athens, KAT Hospital, 145 61 Athens, Greece; dimitriszaxariou@yahoo.com (D.Z.); jvlamis@email.com (J.V.)

**Keywords:** syndesmosis, incisura fibularis, malleolar fractures

## Abstract

Background: Distal tibiofibular injuries are common in patients with malleolar fractures. Malreduction is frequently reported in the literature and is mainly caused by insufficient intraoperative radiological evaluation. In this direction, we performed a prospective observational study to validate the efficacy of the anatomical landmarks of the anterior incisura corner. Methods: Patients with malleolar fractures and syndesmotic instability were reduced according to specific anatomic landmarks and had a postoperative bilateral ankle CT. The quality of the reduction was compared to the healthy ankles. Results: None of the controlled parameters differed significantly between the operated and healthy ankles. Minor deviations were correlated to the normal incisura morphology rather than the reduction technique. Conclusions: The anterior incisura anatomical landmarks can be an efficient way of reducing the distal tibiofibular joint without the need for intraoperative radiological evaluation.

## 1. Introduction

Malleolar ankle fractures are commonly encountered in trauma patients, accounting for 56% of total foot and ankle fractures [1]. Although routinely resulting from low-energy trauma, their impact is not negligible as 52% of patients suffering from these fractures are estimated to develop post-traumatic arthritis at a mean period of 21.1 years [2].

Distal tibiofibular syndesmosis disruption is usually included in the injury sequelae and can affect all four major syndesmotic ligaments [3], resulting in inherent ankle joint instability. For this reason, the traditional classifications of Lauge-Hansen and Weber try to predict the possibility of concomitant syndesmotic injuries and further guide the surgeons towards fixation or not [4,5]. With syndesmotic malreduction and instability negatively affecting both the early and late postoperative periods [6,7], optimal intraoperative assessment is crucial.

There have been many published radiological techniques for intraoperative evaluation of the syndesmotic reduction in the incisura fibularis, ranging from typical measurements on plane views to comparisons to the uninjured contralateral limb [8,9,10]. Nevertheless, none of them has proven significant superiority, simultaneously exposing the patients and the staff to excessive radiological exposure [11]. As a result, the final reduction of the distal tibiofibular syndesmosis remains vulnerable to empirical judgment rather than being an objective finding.

Anatomical landmarks have gained attention in this direction [12,13]. Tornetta et al. found that the incisura landmark can be reliably used to provide an optimal reduction in cadavers [14]. However, this concept has yet to be evaluated when utilized for the fixation of malleolar fractures in patients with concomitant distal tibiofibular disruption. In order to evaluate the efficacy of the incisura corner anatomical landmark, we designed a prospective study in patients with bimalleolar fractures and unstable syndesmosis, which was reduced according to Tornetta et al. principles [14]. We hypothesized that this technique could provide optimal syndesmotic reduction without needing any further radiological evaluation.

## 2. Patients and Methods

We designed a prospective observational study including patients admitted to the authors’ department for the period between 2019 and 2023 suffering from bimalleolar or bimalleolar equivalent fractures and syndesmotic disruption. The exclusion criteria were age under 18, concomitant posterior malleolus fracture, and an injured or previously operated contralateral ankle joint. All participants were evaluated with bilateral ankle computed tomography (CT) in a plantigrade foot position before hospital discharge. The study and methods were approved by our hospital ethics committee.

The patients were informed about the purpose of the study and provided written consent preoperatively. The syndesmotic instability was evaluated during the surgery with the Hook test [15], and only patients with manually established distal tibiofibular disruption were finally enrolled.

### Operative Technique

Patients were operated on in a supine position using a tourniquet under mixed anesthesia according to their age and indications. We performed a typical lateral incision along the distal fibula, slightly anterior to its longitudinal axis and curved anteriorly, distal to the lateral malleolus. After verifying an unstable syndesmosis, we proceeded with the deep dissection of the anterolateral ankle capsule to gain access to the incisura corner. The capsulotomy began from the tibial plafond, curving over the incisura corner and following the lateral malleolus up to the anterior talofibular ligament (ATFL), which remained intact. The ankle joint was irrigated with saline to remove the hematoma and possible intraarticular loose bodies.

**Figure 1:**
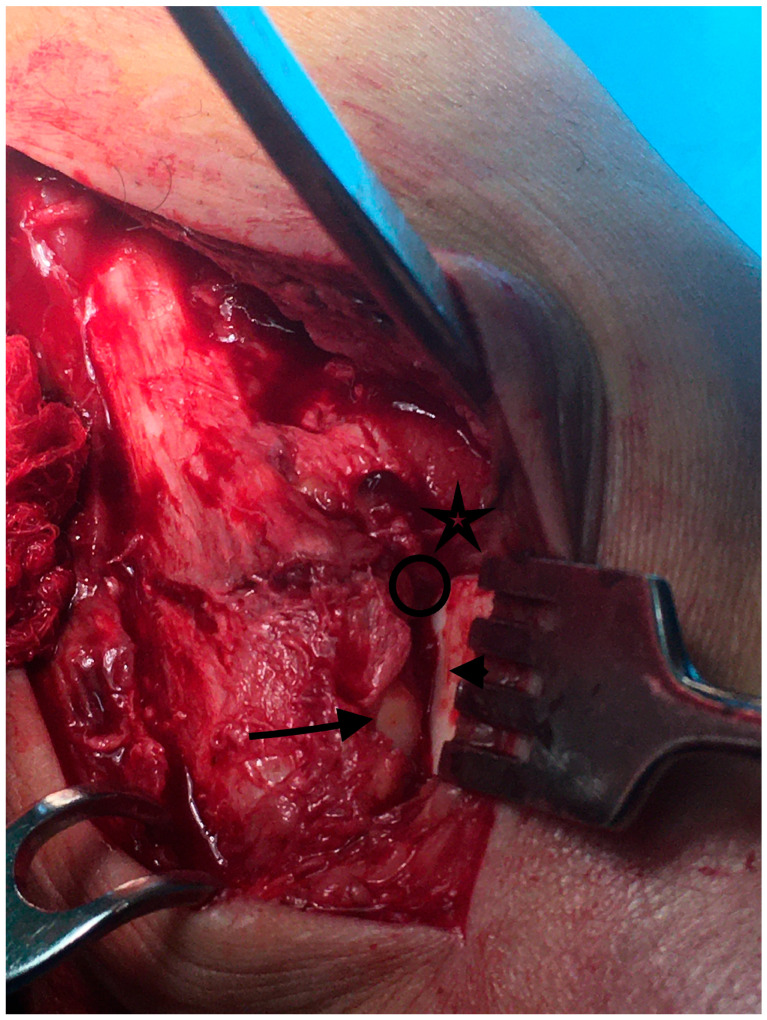
Over the view of the incisura corner after capsulotomy at the initial step, we identify the anterior incisura boarder and the chondral surfaces of the tibial plafond (**✩**), the lateral talar facet (**arrowhead**), and the lateral malleolar facet (**arrow**). Those structures should converge at the incisura corner (**circle**) for optimal reduction.

After remnant soft tissue debridement, the chondral surfaces of the lateral malleolus, tibial plafond, and lateral talus were exposed (Figure 1).

**Figure 2:**
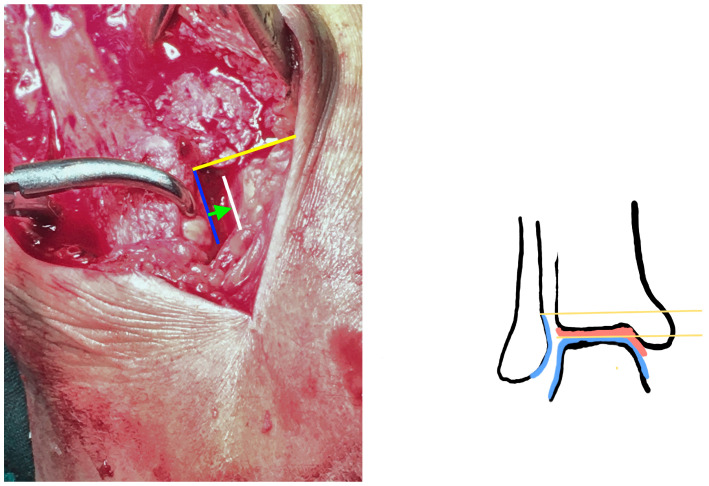
Restoration of the fibular length. The upper part of the lateral malleolus facet (**blue line**) comes in touch with the tibial plafond level (**yellow line**). A shortened fibula would mean that the lateral malleolus facet would be partially inside the incisura fibularis. At that stage, the syndesmosis is not compressed and the lateral talar facet (**white line**) is incongruent from the lateral malleolus (**arrow**). Illustration: Malreduced fibular length. The chondral surface of the lateral malleolus lies above the tibial plafond joint line (distance between two yellow lines).

**Figure 3:**
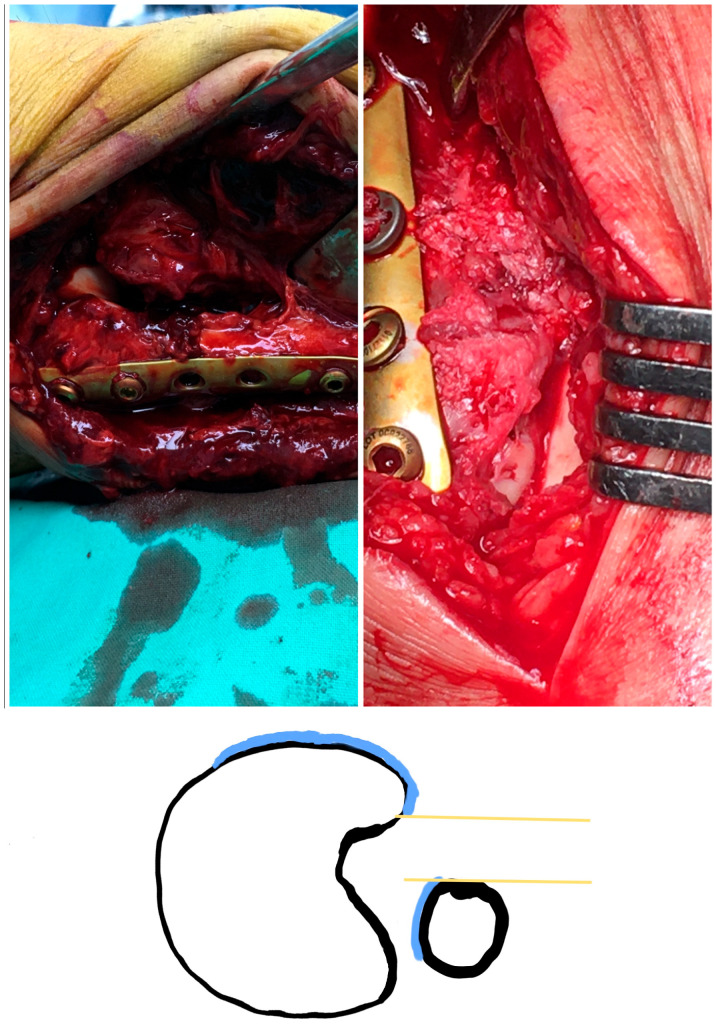
Restoring the anterior fibula translation (sagittal plane) There are two different cases before and after fixation. **Left**: The syndesmosis is widened, and the fibula is posteriorly translated, lower than the anterior incisura boarder. **Right**: The syndesmosis is reduced. There is no gap at the incisura corner, and the lateral malleolus chondral surface is as anterior as the tibial plafond cartilage. **Illustration**: posteriorly malreduced fibula. The chondral surface of the lateral malleolus (fibula blue curve) is posterior to the tibial plafond chondral surface (tibial blue curve), leaving a gap (distance between yellow lines).

The reduction was regarded as anatomical if all the criteria below were met:

1. Anatomical fixation of the fracture site
2. The superior lateral malleolus cartilage was aligned with the anterolateral tibial plafond cartilage (restoring the fibula length) (Figure 2):
3. The lateral malleolus cartilage was put parallel to the lateral talar surface (restoring the fibula rotation)
4. In the sagittal plane, the lateral malleolus cartilage was fixed as anteriorly as the anterolateral tibial plafond cartilage (restoring the sagittal translation) (Figure 3)

Fracture fixation was performed with tubular 1/3 titanium non-locking plates and the positioning of 1 or 2 cortical syndesmotic screws. After the incisura corner debridement, we could efficiently identify the three guiding chondral surfaces. In supination-external rotation (SER) fractures, the 1/3 tubular plate was positioned at first on the distal fragment with two or three distal screws. At that point, we were guided by the anatomical landmarks of the incisura corner to achieve an anatomical reduction; after that, the fracture was provisionally kept in place with a clamp until the final proximal screw positioning. Up to this point, the fibula length and anteroposterior translation had been restored according to our technique. Regarding the rotation, this was reduced so that the lateral malleolus cartilage was parallel to the lateral talar cartilage. An assistant maneuvered the fibula in the desired place during the syndesmotic screw fixation. The screw tightening was done to such a degree that it did not displace the fibula in the incisura corner.

The closure included both the articular capsule and the AITFL, with tight suturing taken care of so that no soft tissue conflict could cause anterolateral impingement. Wound closure was performed in a typical manner. The medial malleolus fractures were consequently fixed as required. All surgeries were performed by the senior author (MR). 

**Figure 4:**
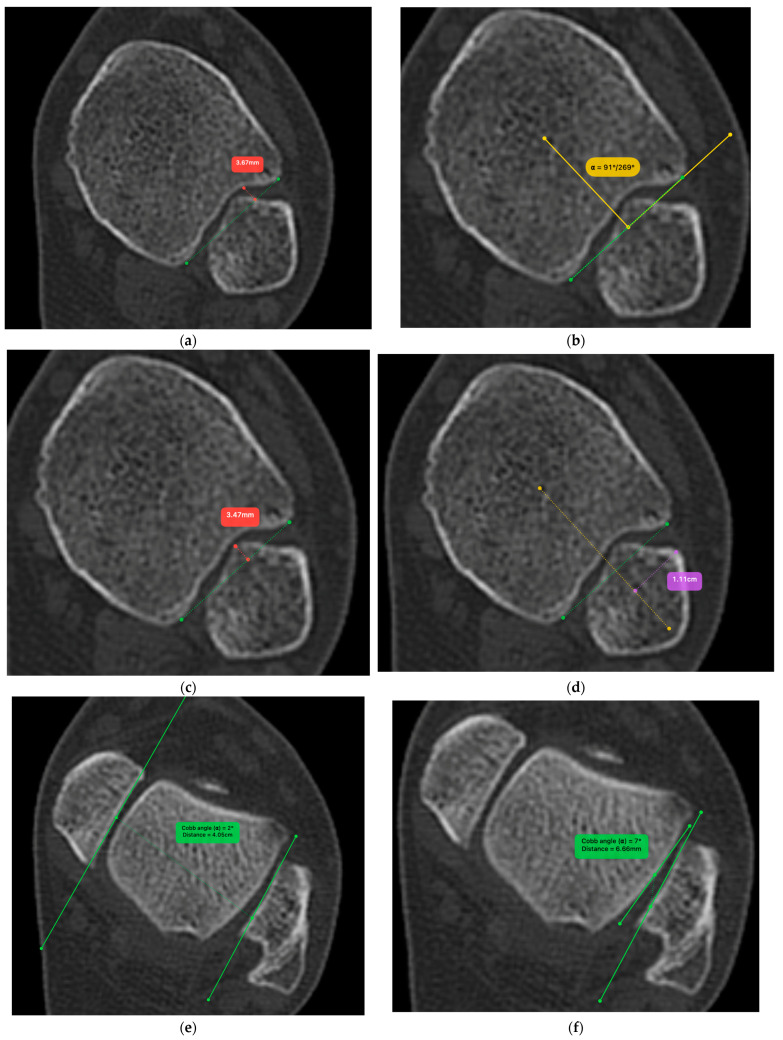

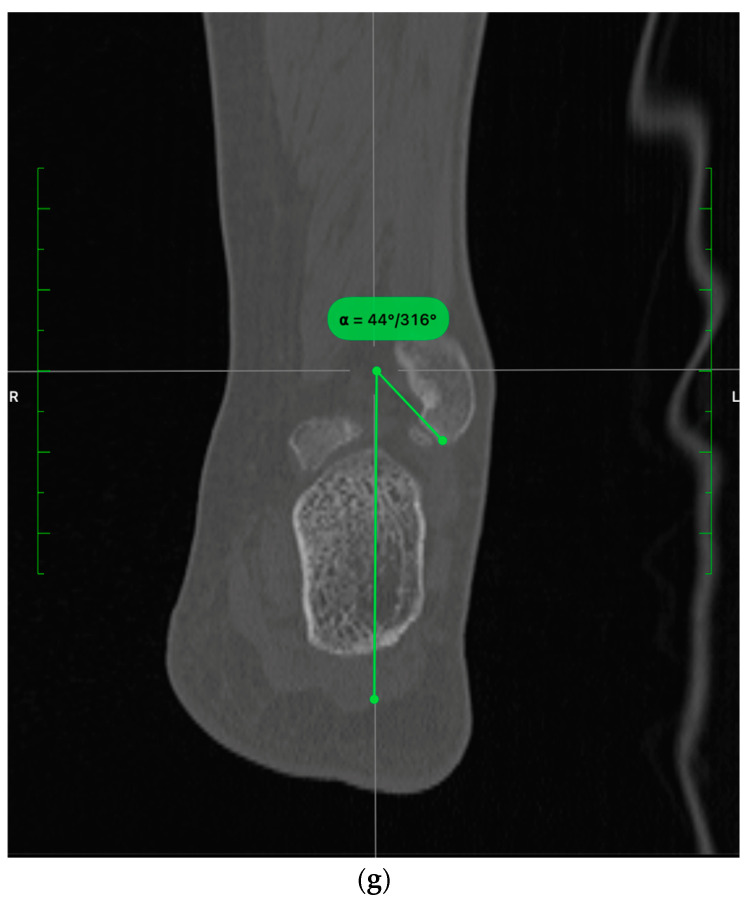
(**a**) Incisura depth: measured as the deepest point from the incisura line; (**b**) Incisura rotation: We first identify the incisura line midpoint and calculate the version from the tibial center; (**c**) Fibular engagement: Calculated as the longest fibular distance from the incisura line; (**d**) Anterior translation: The distance of the most anterior fibular point to the line connecting the tibial center to the incisura midpoint; (**e**) Relative torsion: The angle between the medial and lateral malleoli facets; (**f**) Torsion: The angle between the lateral malleolus and lateral talar facets; (**g**) Measurement of the fibular length. The center of the tibial plafond was drawn along with the tibia longitudinal axis passing from the center. We thereafter identified the most distal part of the fibula. The angle between those two lines was calculated.

The radiological evaluation was done according to Bruzkd et al. [16] by the senior author and included the incisura depth and rotation, fibular engagement, anterior translation, and relative torsion. Proper fibular torsion was additionally measured as the angle between the lateral malleolus facet and the lateral talar cartilage (Figure 4a–f). Finally, we evaluated the fibula length as the angle formed between the most distal point of the lateral malleolus and the center of the tibial plafond (Figure 4g).

The measurements were taken at 10 mm proximally to the tibial plafond. 

The sample statistical analysis was done with a two-tailed *t*-test and Pearson correlation test using SPSS (Version 23). The level of significance for all tests was defined at *p* < 0.05.

## 3. Results

In the four years of our study, 113 patients with malleolar fractures were surgically treated at our clinic. Forty-six patients with a concomitant posterior malleolus (PM) fracture and five with contralateral ankle operations were excluded. From 62 eligible patients with radiologically suspected syndesmotic injury, 49 signed informed consent, and finally, the intraoperative distal tibiofibular instability was validated in 42 of them (85.7%) (Table 1).

The sample consisted of 26 men and 16 women with a mean age of 41.6 years. 48% of the patients had a right ankle, and 52% had a left ankle injury. According to the Lauge-Hansen classification, 73.68% had a supination-external rotation injury, while 26.32% had a pronation-external rotation injury (PER) (Table 2). 

### 3.1. Intraoperative Findings

We identified a torn AITFL in all cases. In 37 of them, there was a mid-substance rupture, 4 had a tibial detachment, and one patient had a concomitant Le Fort fracture. In addition, the proximal fibular fragment was grossly unstable in 13 cases (30.9%), with 11/13 cases being in the PER and only 2/13 in the SER group, respectively. 

Joint debridement after capsulotomy revealed loose chondral bodies from the talar body in only nine patients (21.4%) and were consequently removed. Further inspection was negative for tibial plafond lesions in all patients. In bimalleolar fractures (30/42 cases), the medial malleolus was routinely fixed with one or two partially threaded screws, while the deltoid ligament was transosseous sutured in 2/12 patients (degloving lesion). Of 32 patients with a medial joint inspection, only one (3%) had a superficial medial talar dome chondral injury (Table 3).

### 3.2. CT Evaluation

The mean values of healthy and injured ankles are shown in Table 4. Compared to the uninjured side, the incisura depth difference was measured at 0.48 mm, and the mean rotation difference was 0.06 deg, both non-statistically significant. The fundamental evaluation that validates the proper syndesmotic reduction includes the engagement, anterior translation, torsion, relative torsion, and fibula length. The healthy to injured side difference was 0.22 mm for the fibular engagement, −0.52 mm for the anterior translation, and 1.3 deg for the fibula length. Regarding the rotational reduction, we had a mean difference of −3.4 deg for relative torsion and −3.1 deg for torsion. All parameters above had an insignificant difference (*p* > 0.05). 

The interpretation of these measurements is that, according to the proposed technique, the reduction results in a slightly posterior fibula translation (0.52 mm) with concomitant internal rotation (3.1 deg). Meanwhile, the syndesmosis was not overtightened.

Finally, to further investigate a possible native anatomical relationship of the tibia incisura to the reduction quality, we tried to correlate the incisura rotation and depth to the fundamental parameters. We found a negative correlation between the incisura version and fibula anterior translation (*p* = 0.04) Nevertheless, opposite to what we expected, the Pearson correlation was insignificant between the other measured values meaning that the incisura morphology seems to interfere less with the reduction quality.

## 4. Discussion

We have performed a prospective study on patients with malleolar fractures and concomitant distal tibiofibular instability. The anatomical landmarks along with the reduction sequalae proposed were found to optimally restore the distal tibiofibular syndesmosis in three planes with statistically insignificant changes compared to measurements in healthy ankles.

In the literature, distal tibiofibular joint malreduction is reported in up to 50% of the cases [17] and is directly linked to patient dissatisfaction and progressive arthritic changes [6]. Surgical fixation maneuvers can potentially result in multiplanar malreduction, either from overtightening or poor fracture fixation [18,19]. In addition, although there are several radiological measurements to validate the optimal syndesmotic reduction [20,21], they are prone to subjective interpretation and radiological technique errors [22]. Thus, anatomical landmarks have gained attention in this direction.

The syndesmosis is a complex joint consisting of the distal tibiofibular ligamentous complex and the deep deltoid ligament [23,24]. In a cadaveric study by Mococain et al. [25], the talus turned out to be globally unstable in specimens with dissection of both ligamanetous complexes. On the other hand, the fixation of one of those structures significantly improved the talar stability, while absolute rotational control was established only after restoring both. Additional reports by Grass et al. [26] indicate that the distal tibiofibular joint has a greater impact on controlling the external talar rotation than the lateral shift, with the latter being restricted by the deltoid ligament [27]. Considering those data, we hypothesized that syndesmotic malreduction could not occur in isolation in cases with intact medial structures. Therefore, we excluded patients without a medial malleolus fracture or a deltoid ligament lesion.

The initial analysis started with healthy ankle parameters. Our results agree with the study of Baretta et al. [16] regarding the normal incisura morphology and the positioning of the fibula compared to the incisura, the talus, and the medial malleolus. We added the proper fibula rotation as an additional measurement. In our technique, we tried to place the fibular facet parallel to the lateral talar facet, resulting in a mean external rotation (torsion) of 0.5 deg. Nevertheless, the mean values in the healthy ankles revealed a native internal fibular rotation of 2.58 degrees. Although the difference was not statistically significant, it should be considered during the syndesmotic screw positioning.

In the sagittal plane, the mean healthy to injured anterior fibula translation difference was −0.52 mm (*p* = 0.07), with the tibiofibular compression measured at 0.22 mm (*p* = 0.47). Although there was no overtightening, we had an insignificant posterior fibula translation compared to the uninjured side. This phenomenon could be explained by the native incisura mean retroversion of 3.26 deg, as the fibula tends to subluxate posteriorly. Those two parameters were negatively correlated (*p* = 0.04), but they should be regarded as clinically insignificant since they have no impact on the ankle range of motion [28]. 

**Figure 5:**
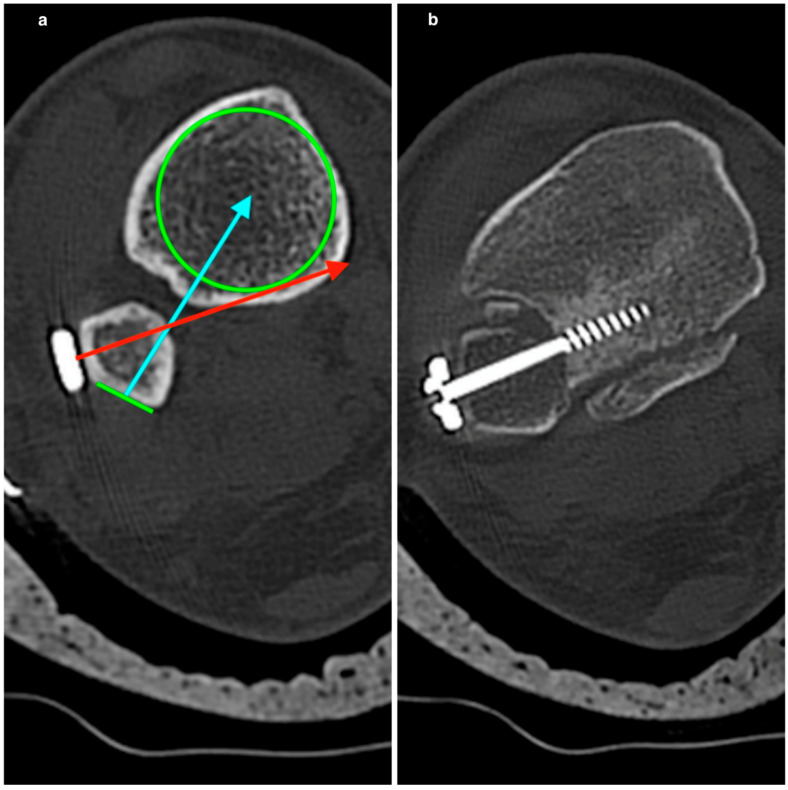
Fibula triangular morphology compromises the optimal center screw positioning. (**a**): In this case, the plate should be placed more posterior LS in order to avoid any possible translational or rotational deformity of the fibula. (**b**): In this case, the direction of the syndesmotic screw misses the tibia center, resulting in posterior fibular translation. Green circle: tibia transverse cut with its potential center. Red arrow: Course of the sydesmotic screw for a plate placed anterolaterally and projectas away of the tibia center. Green line: Supposed plate placed posterolaterally. Blue arrow: Projection of the syndesmotic screw with the plate placed posterolaterally. In this case, the screw can project through the tibia center without rotating or translating the fibula.

After achieving an anatomical reduction of the fracture guided by the incisura corner landmarks, we placed the syndesmotic screw in situ. Unfortunately, this part of the surgery could lead to an unexpected tibiofibular malreduction. The reason for this is the native fibular anatomy and the overtightening phenomenon. In a CT analysis, Baumbach et al. [29] found a continuous shape change of the fibula from proximal to distal. Although they proposed the meta-diaphyseal region as a safe zone for syndesmotic drilling, this can only sometimes be the case. When using 1/3 tubular plates, the positioning depends on the best fitting in each fibular shape, meaning they can be positioned laterally, anteriorly, or posteriorly to the fibula longitudinal axis. This variation does not guarantee that the syndesmotic screw will intersect the center of the tibia, leading to potential rotational and translational fibula malreduction (Figure 5). 

Fibula torsion is another parameter studied, referring to the angle between the medial and lateral malleoli facets. This relationship is crucial to our concept and may be the most important factor when evaluating tibiofibular reduction. Ovaska et al. retrospectively studied the common mistakes leading to malreduced syndesmosis [30]. They found that fibula positioning in the incisura fibularis and the medial malleolus malreductions were the most prominent ones. According to our technique, we restore the torsion using the fibular facet and lateral talar cartilage landmarks, but the relative torsion directly depends on the medial malleolus reduction quality. For this reason, we always try to anatomically reduce the medial malleolus fracture prior to the syndesmotic screw tightening. In cases where we were not satisfied with the incisura corner reduction, we revised medial malleolus fixation (4/42 patients). Although the proposed anatomical landmarks cannot predispose the optimal medial malleolus reduction, they can be used as secondary reassuring means.

Fibula shortening is another possible complication after fracture fixation, resulting in decreased tibial-tibial contact [31,32]. Macroscopically, the optimal fibula length was checked in two ways. At first, we looked for the anatomical reduction of the fibula fracture. After that, we tried to have the lateral malleolar facet proximal chondral surface point at or distal to the tibial plafond chondral surface. A possible shortening of the fibula would mean that the lateral malleolus facet would migrate proximally, so we should correct our reduction. Intraoperatively, though, we did not have any issues regarding the length, as it was easily reduced in cases with simple fracture patterns. In more complex cases, though, we had to be meticulous when evaluating the incisura corner, as the fracture site comminution could compromise the reduction quality. Our results showed no statistical difference in the fibula length between the healthy and injured sites in all patients.

One of the main parts of our technique is the direct inspection and debridement of the ankle joint by performing a lateral capsulotomy. Our rationale is to remove possible osteochondral lesions (OCL) and protect the joint from loose soft tissue that has the potential to cause secondary impingement [33]. Martijn et al. [34] had a systematic review and reported OCL in up to 45% of the patients with ankle fractures, with the vast majority coming from the talus. In addition, Williamson et al. [35] found an even greater rate of up to 54.5% and a positive correlation between the injury pattern and the OCL. In our patient cohort, loose osteochondral bodies were identified in only 21.4% of the patients, all coming from the talus. This mismatch could be explained as those systematic reviews were based on arthroscopic findings. The arthroscopic second view has a greater potential for identifying those injuries, but since it was performed several weeks after the fracture fixation, one cannot be sure if those OCLs were caused by the fracture or by secondary impingement. Soft tissue impingement can also occur in this patient cohort [36]. In our experience, the primary reason is the prolapsing anterolateral capsule, including the AITFL remnants. We believe this can be avoided with meticulous suturing of both structures. Of course, scar formation is somehow unavoidable, especially in cases with residual instability of the talus.

Our study has several limitations. At first, we described the incisura retroversion by considering the mean values. It is essential to underline that we had patients with neutral or even anteverted morphology. Thus, the hypothesis that the fibula is usually subluxated posteriorly cannot be exact for each patient separately. Similar conclusions should involve normal fibula torsion and relative torsion as well.

Our measurements were taken 10 mm proximally to the tibial plafond in all patients. The tibia length, though, could be a significant weakness of the study as the appropriate mid-incisura point could be higher or lower than this distance [37].

We have excluded patients with a posterior malleolus fracture. Our surgical approach is more anterior than usual, and we cannot access the posterior malleolus fragment for direct fixation. Since the PM can involve the posterior part of the incisura fibularis, malreduction would alter the morphologic measurements, misleading the interpretation of the tibiofibular reduction quality.

We have intentionally not included our technique’s clinical impact and score assessment. Our goal was to investigate the role of specific anatomical landmarks in optimal syndesmotic reduction. From this aspect, we cannot speculate on how to achieve better clinical results by following this algorithm. However, we know we are significantly closer to the normal ankle anatomy.

Finally, our study lacks power. This is a significant weakness, meaning that our statistically insignificant results could be biased and need further validation in a larger patient cohort.

## 5. Conclusions

The anatomical landmarks of the anterior incisura corner can reliably predict the anatomical distal tibiofibular joint reduction in patients with bimalleolar and bimalleolar equivalent fractures. The proposed technique’s results are reproducible and efficient and avoid excessive intraoperative radiological exposure. Nevertheless, we need to be cautious with the results’ interpretation because of the lack of study power, and we need further studies to evaluate their clinical impact.

## Figures and Tables

**Table 1:** Study flowchart and final patient selection.

113	Patients operated for malleolar fractures 46 excluded due to posterior malleolus fx 5 excluded due to contralateral side past operation
62	13 patients did not consent 7 patients were excluded beacuse the syndesmosis instability was not verified intraoperatively
42	Total number of patients included in the study

**Table 2:** Sample demographics and injury patterns.

Gender	23M/16F
Mean Age	41.6 years [Range: 19–71]
Injury Mechanism	SER: 73.68% PER: 26.32%

**Table 3:** Intraoperative findings. IA: Intra articular.

Torn AITFL	42/42 (100%)
Midsubstance rupture	37/42 (88%)
Tibial insertion	4/42 (9%)
Fibular insertion	1/42 (3%)
**Gross proximal fragment instability**	13/42 (30.9%)
SER	2/13
PER	11/13
**IA loose bodies**	9/42 (21.4%)

**Table 4:** Mean values of the basic parameters as measured in the CT scans.

	Incisura Depth (mm)	Incisura Rotation (deg)	Fibular Engagement (mm)	Fibula Anterior Translation	Fibula Length (deg)	Relative Torsion (deg)	Torsion (deg)
Healthy side	4.08	−3.26	0.81	8.8	50.6	9.35	−2.58
Injured side	3.6	−3.33	0.59	9.33	49.2	12.8	0.5
**Side to side mean Difference**	**0.48**	**0.06**	**0.22**	**−0.52**	**1.3**	**−3.4**	**−3.1**
*p*	>0.05						

## Data Availability

Not available.
